# The inherent defects of cold atmospheric plasma for the treatment of burns

**DOI:** 10.3389/fphar.2025.1540704

**Published:** 2025-02-25

**Authors:** Yingying Li, Hong Guan, Guojuan Fan, Jinlong Ma

**Affiliations:** ^1^ Hebei Chemical and Pharmaceutical College, Shijiazhuang, Hebei, China; ^2^ School of Agriculture and Forestry Technology, Weifang Vocational College, Weifang, Shandong, China; ^3^ Dermatology, Weifang Hospital of Traditional Chinese Medicine, Shandong Second Medical University, Weifang, Shandong, China; ^4^ School of Pharmacy, Shandong Second Medical University, Weifang, Shandong, China

**Keywords:** cold atmospheric plasma, burns treatment, inherent defects, UV radiation, high flow rate gas

## Introduction

The skin system constitutes the human body’s largest organ, acting as a robust shield against various harmful invaders, including microbes and ultraviolet (UV) rays (Ling et al., 2021; Cheng et al., 2022). When the skin is burned, its barrier function is compromised, making the affected areas susceptible to microbial influence, which can lead to a series of pathological changes in the body. The process of wound recovery from burns can be categorized into three distinct phases: the inflammatory response, tissue proliferation, and the tissue remodeling of the affected area (Qi et al., 2024). A multitude of bacteria multiply rapidly in the compromised area, during the inflammatory phase (1–2 days after burn), which in turn slows down the wound’s recovery due to the formation of ulcers (Iacob et al., 2020). During the tissue proliferation phase, which typically occurs 1–2 weeks post-burn, the wound site is marked by the influx and activation of fibroblasts, as well as the development of new blood vessels, which are crucial for the healing process (Wietecha et al., 2020). The tissue remodeling phase initiates approximately 2 weeks following skin injury and continues for a number of weeks, aimed at restoring the skin’s physiological functionality (Wang et al., 2022). This stage is primarily characterized by the metamorphosis of granulation tissue into a more advanced connective tissue. However, an overproduction of collagen during this phase can readily result in the formation of scars. Therefore, the development of a burn treatment system that possesses antibacterial properties, promotes skin repair, and inhibits scar formation is of great significance.

Over the past few years, there has been a growing interest in the versatile potential of cold atmospheric plasma (CAP) across various fields, such as sterilization and disinfection, dental cleaning, cosmetology, and the treatment of skin diseases, trauma, and cancer, attracting widespread attention in the biomedical field (Chen et al., 2021; Lunov et al., 2016; Qin et al., 2022). This is mainly due to its rich content of physiologically active components, such as reactive free radicals (ROS and RNS, etc.), charged particles (positive and negative), electrons, and ultraviolet light (Chen et al., 2021; Szili et al., 2021). Research has found that CAP not only has antibacterial effects but also can accelerate the healing process of wounds through the stimulation of fibroblast proliferation and angiogenesis, making it a potential ideal method for treating burns (Dijksteel et al., 2020; Boekema et al., 2021; Oliver et al., 2024; Bagheri et al., 2023; Plattfaut et al., 2021; Duchesne et al., 2019; Nastuta et al., 2011; Bhartiya et al., 2021; Frescaline et al., 2020). The applicant’s previous work in combining CAP with nanomedicine for the treatment of third-degree burns has also proven that CAP has the functions of antibacterial activity and skin repair (Wang et al., 2023).

As a new type of treatment, most of the CAP devices currently used are self-built in laboratories, with significant differences in parameters and operations between different devices, resulting in inconsistent dosage and frequency of treatment. In contrast, in our research, we found that CAP has other inherent defects that severely limit its application in burn treatment.

## UV radiation

Although some research reports indicate that the risks are minimal under specific conditions (Lotfi et al., 2024), there is also literature suggesting that long-term treatment can trigger cytotoxic effects and reduce cell viability (Sremački et al., 2021). In the absence of a protective medium, vacuum ultraviolet/ultraviolet radiation significantly contributes to plasma - induced DNA damage and cytotoxicity (accounting for 70%). While normal skin can mitigate the harm of UV light through the stratum corneum, burned areas lack this protective barrier (Shimizu et al., 2010). The damage caused by UV radiation to the skin is mainly manifested as: (1) UV light can impair the physiological functions of macromolecules within cells (proteins, lipids, and nucleic acids, etc.), inducing cellular damage (Gu et al., 2020); (2) It also destroys collagen and elastic fibers, accelerating skin aging and slowing down wound healing (Xiao et al., 2022); (3) By forming pyrimidine dimers and other DNA damages, it leads to skin cancer (Saha et al., 2020; Douki et al., 2024). These hazards of UV radiation limit the drug dosage and administration frequency of CAP treatment, thereby affecting the speed of wound recovery. Although existing literature has noted the hazards of UV light in CAP and improved CAP devices to suppress the generation of UV light, it has also significantly weakened the intensity of its active free radicals (Shimizu et al., 2010). Therefore, how to effectively filter out UV light while retaining the active free radicals of CAP remains a challenge.

There are two potential approaches to address this issue. The first method involves utilizing an instrument that leverages the linear propagation characteristics of light to filter out the majority of UV light. However, this approach may, to some extent, diminish the quantity of active ions that reach the target site. The second idea is to develop an adjuvant drug capable of absorbing UV light, akin to a sunscreen. It is crucial to ensure that the selected sunscreen does not react with ROS or RNS, as such reactions could compromise the overall activity of CAP.

## High flow rate gas

A high flow rate gas of CAP can make the wound area extremely dry (Dejonckheere et al., 2024; Shaitelman et al., 2015), which contradicts the theory of moist wound healing and hinders wound healing, preventing it from fully exerting its functions of promoting wound healing and inhibiting scarring. A moist wound environment facilitates the natural process of autolytic debridement, alleviates pain, minimizes scarring, stimulates collagen production, and encourages the migration of keratinocytes across the wound bed, thus collectively contributing to an enhanced wound healing process (Nuutila and Eriksson, 2021). On the other hand, a dry wound environment has at least four disadvantages: (1) Excessive dryness of the wound can dehydrate the traumatized tissue, leading to further tissue damage and the formation of unfavorable eschar, making it difficult for new epithelial cells to move, and prolonging the wound healing time (Breuing et al., 1992); (2) The wound is more susceptible to infection (Daly et al., 2016); (3) Larger scars are produced (Junker et al., 2013); (4) It intensifies the patient’s pain (Bechert and Abraham, 2009). Therefore, when using CAP for burn treatment, another issue that needs to be addressed is the dryness brought about by the high flow rate of gas.

Certainly, it is feasible to strike a balance between the efficacy of CAP and the minimization of dryness by fine-tuning the gas flow rate threshold. Nevertheless, this equilibrium merely replicates the wound environment akin to that of exposure therapy and falls considerably short of attaining the optimal state of wet wound healing. In light of this, a question emerges: could we integrate hydrogel, hydrosol, and other relevant materials with CAP to actualize a dual benefit of wet wound healing and combined CAP treatment?

## Discussion

In this Opinion, we have summarized the inherent defects that severely limit CAP application in burn treatment. Most of the studies reported self-built in laboratories, with significant differences in parameters and operations between different devices, resulting in inconsistent dosage and frequency of treatment, but ultraviolet light and high-speed gas flow are rarely performed. Therefore, how to effectively address the issues of UV and high-speed gas flow in CAP is a fundamental problem that needs to be solved for the use of CAP in burn treatment.

In view of the challenges associated with UV radiation, innovative instruments could be devised to effectively filter out ultraviolet rays. Additionally, CAP adjuvant drugs might be developed, leveraging the linear propagation traits of light to absorb ultraviolet radiation. When it comes to the drying issue resulting from high-flow-rate gas, the design and application of hydrogels, hydrosols, and other related substances could be explored to facilitate wet wound healing. This Opinion piece offers a novel research vantage point for the utilization of CAP in burn treatments, carrying significant theoretical and practical implications for the exploration of new therapeutic approaches in the field of burns.
